# Periodontal disease and risk of benign prostate hyperplasia: a cross-sectional study

**DOI:** 10.1186/s40779-019-0223-8

**Published:** 2019-11-13

**Authors:** Lan Wu, Bing-Hui Li, Yun-Yun Wang, Chao-Yang Wang, Hao Zi, Hong Weng, Qiao Huang, You-Jia Zhu, Xian-Tao Zeng

**Affiliations:** 1 Center for Evidence-Based and Translational Medicine, Zhongnan Hospital of Wuhan University, Wuhan, 430071 China; 2 Department of Stomatology, Zhongnan Hospital of Wuhan University, Wuhan, 430071 China; 3 Center for Evidence-Based Medicine, Institute of Evidence-Based Medicine and Knowledge Translation, Henan University, Kaifeng, 475000 China; 4 Department of Urology, Zhongnan Hospital of Wuhan University, Wuhan, 430071 China

**Keywords:** Benign prostate hyperplasia, Periodontal disease, Periodontitis, Risk factor, Inflammatory disease

## Abstract

**Background:**

Both periodontal disease and benign prostatic hyperplasia are age-related diseases that affect millions of people worldwide. Hence, this study aimed to investigate the association between periodontal disease and the risk of benign prostatic hyperplasia.

**Methods:**

A total of 4930 participants were selected from an available health examination that was carried out in 2017, only males were considered for further analysis. All eligible males were divided into benign prostatic hyperplasia and normal groups, the benign prostatic hyperplasia group was then divided into prostate volume ≤ 60 g and > 60 g subgroups; all their periodontal status was extracted and then into normal (CPI score of 0), periodontal disease (CPI score between 1 and 4), and periodontitis (CPI score between 3 and 4) groups. The correlation between periodontal disease and benign prostatic hyperplasia was investigated using logistic regression analyses and greedy matching case-control analysis. Subgroup analysis based on prostate volume was also performed. All analyses were conducted with SAS 9.4 software.

**Results:**

A total of 2171 males were selected for this analysis. The presence of periodontal disease significantly increased the risk of benign prostatic hyperplasia by 1.68 times (*OR* = 1.68, 95% CI: 1.26–2.24), and individuals with periodontitis showed a higher risk (*OR* = 4.18, 95% CI: 2.75–6.35). In addition, among matched cases and controls, this association remained robust (periodontal disease: *OR* = 1.85, 95% CI: 1.30–2.64; periodontitis: *OR* = 4.83, 95% CI: 2.57–9.07). Subgroup analysis revealed that periodontal disease significantly increased benign prostate hyperplasia risk as well (for prostate volume ≤ 60 g: *OR* = 1.64, 95% CI: 1.22–2.20; for volume > 60 g: *OR* = 2.17, 95% CI: 1.04–4.53), and there was a higher risk in the group with a prostate volume greater than 60 g.

**Conclusion:**

Periodontal disease is significantly and positively associated with an increased risk of benign prostatic hyperplasia. Further validation studies should be performed to explore the relationship between periodontal treatment and benign prostate hyperplasia.

## Background

Periodontal disease is a complex polymicrobial inflammation and a global burden disease (GBD), and periodontal disease mainly includes gingivitis and periodontitis. In 2015, the incidence of severe chronic periodontitis reached 616 million cases around the world [1]. In China, the standardized disability-adjusted life years (DALYs) rate for this disease has risen from 24.7 in 1990 to 25.7 in 2013 according to the 2013 GBD study [2]. Moreover, periodontal disease is involved in increasing the risk of various systematic diseases, such as atherosclerotic complications (cardiovascular disease [3, 4], arterial stiffness [5], carotid intima-media thickness [6], carotid atherosclerosis [7], stroke [8], coronary heart disease [9], erectile dysfunction [10], hypertension [11, 12], etc.), cancers (head and neck cancer [13], breast cancer [14], lung cancer [15], pancreatic cancer [16], etc.), and metabolic diseases (diabetes [17], overweight/obesity [18], gestational diabetes mellitus [19], etc.).

Benign prostatic hyperplasia (BPH) is defined as unregulated proliferation of connective tissue, smooth muscle and glandular epithelium within the prostate transition zone and is one of the most common diseases in humans. It is estimated that the doubling time of BPH growth is 4.5 years among individuals between the ages of 31 and 50 and 10 years among those between 51 and 70 years old [20]. A meta-analysis including data from 25 countries showed a lifetime prevalence of BPH of 26.2% [95% confidence interval (CI): 22.8–29.6%] without regional or ethnic discrepancies [21]. BPH treatment imposes heavy social and economic burdens on individuals, families, communities and countries. In addition, the estimated cost for annual BPH treatment is approximately $4 billion in the United States alone [22]. Hence, identifying risk factors for BPH to improve early prevention is meaningful.

Periodontal disease and BPH shared common risk factors, such as age, smoking, obesity, diabetes, physical activity, socioeconomic status, and inflammation. Accordingly, it has been hypothesized that periodontal disease may be a marker for BPH. In 2013, Boland et al. performed a case-control study and, for the first time, found that periodontal disease could significantly increase the risk of BPH by 1.50 times [odds ratio (*OR*) = 1.50, 95% CI, 1.05–2.10] [23]. In 2017, Estemalik et al. demonstrated a marked positive association between oral pathogens and BPH onset; that is, at least one oral pathogen could be detected in 9 out of 10 BPH patients in their prostatic secretions [24]. Therefore, we performed the current study to explore whether there exists an association between periodontal disease and BPH in the Chinese Han population.

## Methods

We used the Strengthening the Reporting of Observation Studies in Epidemiology (STROBE) [25] statement to report this cross-sectional study.

### Study design and data extraction

With a retrospective design, this study adopted all available data from a health examination at Henan University in 2017 [12]. This study was reviewed and approved by the Committee for Ethical Affairs of the Huaihe Hospital of Henan University, Henan Province. We first identified 4930 records with full information on name, gender, date of birth, relevant physical examinations and laboratory examinations on February 12, 2019, and finished analyses on April 25, 2019. The records would be included for further analysis when information on gender, age, periodontal status, weight, height and prostate status was all included. Only data for males were selected for further screening. However, the health examination did not refer to the history of alcohol or tobacco use, and the absence of these data could result in insufficient capability to assess confounding factors. All eligible data were finally divided into BPH Yes and No groups according to clinical diagnosis results accompanied by relevant examination information, including physical examination and prostate ultrasonography [26]. Patients with records of receiving prostatectomy were automatically enrolled into the BPH group.

### Assessment of variables

The largest anteroposterior (height, H), transverse (width, W), and cephalocaudal (length, L) prostate diameters were detected for BPH patients using transabdominal B-ultrasonography. A normal prostate was directly recorded without information on those measurements. Prostate volume (PV) was obtained using the prostate ellipsoid formula: PV = π/6 × [H (cm) × W (cm) × L (cm)]. Then, the BPH group was divided into PV ≤60 g and > 60 g subgroups. Clinical oral examinations of the participants in the dental chair were performed by dentists using a headlamp, a mouth mirror, and a periodontal probe. Periodontal status was assessed using the Community Periodontal Index (CPI), and maximal CPI score of sextants ranged from 0 to 4: 0 referred to healthy periodontal tissue, 1 to the appearance of bleeding, 2 to the existence of calculus, 3 to pocket depth of 4–5 mm, and 4 to pocket depth ≥ 4 mm [27, 28]. A CPI score between 1 and 4 denoted an unhealthy periodontium, while a score of 0 referred to a healthy periodontium. For data analysis, based on periodontal status, individuals were divided into normal (CPI score of 0), periodontal disease (CPI score between 1 and 4), and periodontitis (CPI score between 3 and 4) groups.

Covariates were selected based on originally recorded data and the current knowledge of potential influencers of BPH and periodontal disease. The weight and height of each participant were measured, and body mass index (BMI) was calculated as weight in kilograms divided by height in meters squared (kg/m^2^). Systolic blood pressure (SBP; mmHg) and diastolic blood pressure (DBP; mmHg) were measured, and hypertension referred to SBP ≥140 mmHg, DBP ≥90 mmHg or use of medicine for hypertension. Covariates for the analyses included age, BMI, hypertension status, fasting blood glucose (FBG, ng/ml), serum lipid composition [total cholesterol (TC; mmol/L), triglycerides (TGs; mmol/L), high-density lipoprotein cholesterol (HDL-C; mmol/L), low-density lipoprotein cholesterol (LDL-C; mmol/L)], erythrocyte sedimentation rate (ESR; mm/h), C-reactive protein (CRP; mg/L), urea nitrogen (UN; mmol/L), uric acid (UA; μmol/L), creatinine (μmol/L), and proteinuria.

### Statistical analysis

Basic characteristics were summarized for the overall sample as well as for the normal and BPH groups. Categorical variables are shown as counts (percentages), while continuous variables are shown as the means ± standard deviations or medians (interquartile ranges) based on results from the normal distribution test. Comparisons between the normal and BPH groups were conducted using two independent sample *t* tests (or Wilcoxon rank sum tests) for continuous variables and chi-squared tests (or Fisher exact tests) for categorical variables.

The association between periodontal diseases and BPH was examined with univariable and multivariable logistic regressions. Further and detailed evaluations were performed according to the CPI scores individually. Three models were established stepwise. Specifically, model 1 was constructed without adjustment; model 2 was adjusted for age, BMI, and blood pressure status; and model 3 adopted additional adjustments for FBG, TC, TGs, LDL-C, HDL-C, ESR, UN, UA, creatinine, and proteinuria in addition to the variables adjusted in model 2. Statistical presentation includes *OR*, 95% CI and corresponding *P* value for simplification.

Sensitivity analysis was performed to detect whether differences in age, BMI, or blood pressure status between the normal and BPH groups influenced the final results. We matched cases and controls using greedy matching age ± 5 years, BMI ±2, and the same blood pressure status (hypertension and normotension). If the results of the sensitivity analysis were similar to the original results, associations were robust; otherwise, we adopted matched results. Subgroup analysis was conducted on the basis of PV to explore specific effects in groups with different PV sizes. All statistical analyses were calculated with SAS 9.4 software. Tests on statistical significance were two-sided, and *P* < 0.05 was considered significant.

## Results

### General characteristics

**Fig. 1:**
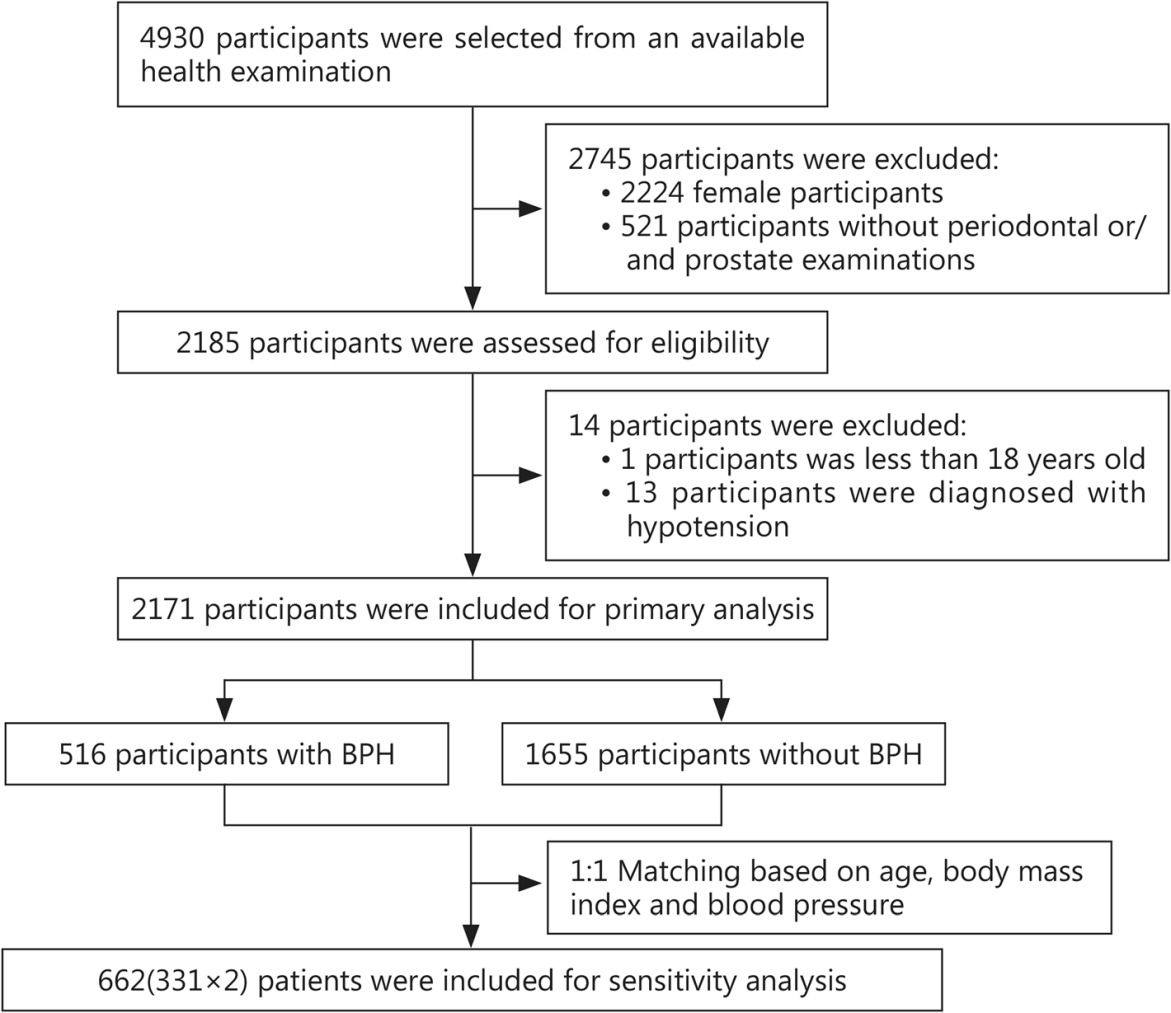
Summary the selection process. A total of 2171 participants were included for primary analysis, from which 516 were classified into the BPH group. About 662 patients were included for sensitivity analysis

Finally, 2171 participants were included in this analysis after excluding females and those not undergoing periodontal or prostate status examinations. The selection process is shown in Fig. 1. Of the participants, 516 were classified into the BPH group, while 1655 were classified into the normal group. Moreover, 1405 subjects were free from periodontal disease, while 766 had periodontal disease, accounting for 35.3% of all participants. The mean age was 51.09 ± 15.25 years in the overall sample, 67.02 ± 12.83 years in the BPH group and 46.13 ± 12.25 years in the normal group. The mean BMI was 24.73 ± 3.08 kg/m^2^ in the BPH group and 25.20 ± 3.00 kg/m^2^ in the normal group; the mean PV was 41.03 ± 18.11 g in the BPH group. Age, BMI and blood pressure status showed statistically significant differences between the two groups (*P* < 0.05). The baseline characteristics of all of these participants are presented in Table 1.

**Table 1:** Baseline characteristics of the participants (*n =* 2171)

Characteristics	Sample (*n =* 2171)	Benign prostatic hyperplasia		*P*
		Yes (*n =* 516)	No (*n =* 1655)	
Age (year, mean ± SD)	51.09 ± 15.25	67.02 ± 12.83	46.13 ± 12.25	0.00
BMI (kg/m^2^, mean ± SD)	25.09 ± 3.03	24.73 ± 3.08	25.20 ± 3.00	0.00
Blood pressure [*n*(%)]				0.00
Hypertension	794 (37.4)	249 (49.3)	545 (33.7)	
Normotension	1327 (62.6)	256 (50.7)	1071 (66.3)	
Periodontal disease [*n*(%)]				0.40
No	1405 (64.7)	342 (66.3)	1063 (64.2)	
Yes	766 (35.3)	174 (33.7)	592 (35.8)	
FBG (ng/ml, mean ± SD)	5.64 ± 1.45	5.96 ± 1.62	5.54 ± 1.37	0.00
LDL-C (mmol/L, mean ± SD)	2.77 ± 0.70	2.75 ± 0.72	2.78 ± 0.69	0.43
Triglycerides (mmol/L, mean ± SD)	1.55 ± 1.01	1.39 ± 0.92	1.61 ± 1.04	0.00
HDL-C (mmol/L, mean ± SD)	1.23 ± 0.25	1.23 ± 0.25	1.23 ± 0.26	0.86
TC (mmol/L, mean ± SD)	4.63 ± 0.86	4.63 ± 0.91	4.63 ± 0.85	0.89
ESR (mm/h, mean ± SD)	5.51 ± 6.01	8.24 ± 8.36	4.66 ± 4.74	0.00
Uric acid (μmol/L, mean ± SD)	320.44 ± 72.01	314.48 ± 71.63	322.31 ± 72.06	0.03
Creatinine (μmol/L, mean ± SD)	81.25 ± 13.04	80.03 ± 14.13	81.64 ± 12.65	0.02
Urea nitrogen (mmol/L, mean ± SD)	5.13 ± 1.21	5.20 ± 1.39	5.10 ± 1.14	0.13
Prostate volume (g, mean ± SD)		41.03 ± 18.11	NR	NA
≤ 60 g (*n* = 444)		35.93 ± 9.24	NR	
> 60 g (*n* = 62)		77.92 ± 22.79	NR	

*BMI* Body mass index, *FBG* Fasting blood glucose, *TC* Total cholesterol, *LDL-C* Low-density lipoprotein cholesterol, *HDL-C* High-density lipoprotein cholesterol, *ESR* Erythrocyte sedimentation rate, *NR* Not reported, *NA* Not available

### Overall analysis

Table 2 shows the overall results from the univariable and multivariable logistic regression analyses. In the univariable analysis, periodontal disease was not related to BPH risk (*OR* = 0.92, 95% CI: 0.74–1.13), but it was shown that periodontitis might increase the risk of BPH (*OR* = 4.97, 95% CI: 3.59–6.90). Adjusted analysis using model 2 revealed that periodontal disease (*OR* = 1.40, 95% CI: 1.15–1.69) and periodontitis (*OR* = 4.10, 95% CI: 2.75–6.09) were both significantly associated with an increased risk of BPH (*P* < 0.05). Adjusted analysis adopting model 3 also achieved similar results (periodontal disease: *OR* = 1.68, 95% CI: 1.26–2.24; periodontitis: *OR* = 4.18, 95% CI: 2.75–6.35; *P* < 0.05).

**Table 2:** Multivariable analysis results for the relationship between periodontal disease and benign prostatic hyperplasia

Category	Periodontal disease		Periodontitis	
	*OR* (95% CI)	*P*	*OR* (95% CI)	*P*
Overall				
Model 1	0.92 (0.74–1.13)	0.43	4.95 (3.59–6.90)	< 0.00
Model 2	1.75 (1.34–2.33)	< 0.00	4.10 (2.75–6.09)	< 0.00
Model 3	1.68 (1.26–2.24)	< 0.00	4.18 (2.75–6.35)	< 0.00
Prostate volume ≤ 60 g				
Model 1	0.94 (0.75–1.18)	0.59	5.08 (3.62–7.13)	< 0.00
Model 2	1.73 (1.30–2.31)	< 0.00	4.13 (2.75–6.19)	< 0.00
Model 3	1.64 (1.22–2.20)	< 0.01	4.20 (2.74–6.43)	< 0.00
Prostate volume > 60 g				
Model 1	0.74 (0.42–1.29)	0.29	4.18 (2.04–8.57)	< 0.00
Model 2	2.26 (1.10–4.65)	0.03	7.32 (2.90–18.48)	< 0.00
Model 3	2.17 (1.04–4.53)	0.04	8.36 (3.21–21.78)	< 0.00

*OR* Odds ratio, *CI* Confidence interval, Model 1: Without adjustment; Model 2: Adjusted for age, body mass index and blood pressure status; Model 3: Further adjusted for fasting blood glucose, low-density lipoprotein cholesterol, high-density lipoprotein cholesterol, triglycerides, total cholesterol, erythrocyte sedimentation rate, uric acid, creatinine, and urea nitrogen

### Subgroup analysis

Table 2 also demonstrates the subgroup results from univariable and multivariable logistic regression analyses. Periodontal disease was not associated with BPH risk in either the PV ≤60 g group or the PV > 60 g group (*P* > 0.05). However, periodontitis was significantly associated with an increased risk of BPH in both groups (*P* < 0.05). After adjustment, both model 2 and model 3 analyses revealed a significant positive relationship of periodontal disease with BPH onset (*P* < 0.05). However, the disease risk in the PV > 60 g group was higher than that in the PV ≤60 g group.

### Sensitivity analysis

We matched cases and controls using greedy matching age ± 5 years, BMI ±2, and the same blood pressure status (hypertension and normotension). Finally, 662 participants were enrolled in the analysis, involving 331 cases and 331 controls. Table 3 presents basic information for overall subjects as well as for cases and controls. The results from the model 3 analysis indicated that periodontitis (*OR* = 4.83, 95% CI: 2.57–9.07) and periodontal disease (*OR* = 1.85, 95% CI: 1.30–2.64) significantly increased BPH susceptibility. Compared to the overall analysis findings, these results demonstrated that the association of periodontal disease with BPH was robust.

**Table 3:** Baseline characteristics of the matched participants (*n =* 662)^a^

Characteristics	Samples (*n =* 662)	Benign prostatic hyperplasia		*P*
		Yes (*n =* 331)	No (*n =* 331)	
Age (year, mean ± SD)	61.66 ± 11.41	61.92 ± 11.57	61.41 ± 11.25	0.57
BMI (kg/m^2^)	24.82 ± 2.97	24.85 ± 2.96	24.79 ± 2.99	0.80
Blood pressure [*n*(%)]				1.00
Hypertension	320 (48.3)	160 (48.3)	160 (48.3)	
Normotension	342 (51.7)	171 (51.7)	171 (51.7)	
Periodontal disease [*n*(%)]				0.00
No	459 (69.3)	208 (62.8)	251 (75.8)	
Yes	203 (30.7)	123 (37.2)	80 (24.2)	
FBG (ng/ml, mean ± SD)	5.92 ± 1.63	5.84 ± 1.48	5.99 ± 1.76	0.25
LDL-C (mmol/L, mean ± SD)	2.83 ± 0.73	2.80 ± 0.72	2.85 ± 0.73	0.40
Triglycerides (mmol/L, mean ± SD)	1.46 ± 0.90	1.45 ± 0.98	1.47 ± 0.80	0.69
HDL-C (mmol/L, mean ± SD)	1.24 ± 0.25	1.22 ± 0.23	1.26 ± 0.27	0.09
TC (mmol/L, mean ± SD)	4.73 ± 0.91	4.67 ± 0.90	4.78 ± 0.93	0.13
ESR (mm/h, mean ± SD)	6.75 ± 6.53	6.48 ± 6.29	7.02 ± 6.77	0.29
Uric acid (μmol/L, mean ± SD)	313.41 ± 73.87	313.15 ± 68.88	313.68 ± 78.68	0.93
Creatinine (μmol/L, mean ± SD)	79.85 ± 14.16	79.74 ± 13.15	79.97 ± 15.13	0.83
Urea nitrogen (mmol/L, mean ± SD)	5.19 ± 1.41	5.11 ± 1.34	5.26 ± 1.48	0.17
Prostate volume (g, mean ± SD)		39.56 ± 17.21	NR	NA
≤ 60 g (*n* = 296)		35.53 ± 9.19	NR	
> 60 g (*n* = 32)		77.40 ± 26.20	NR	

*BMI* Body mass index, *FBG* Fasting blood glucose, *TC* Total cholesterol, *LDL-C* Low-density lipoprotein cholesterol, *HDL-C* High-density lipoprotein cholesterol, *ESR* Erythrocyte sedimentation rate, *NR* Not reported, *NA* Not available

^a^We matched cases and controls using greedy matching age ± 5 years, BMI ±2, and same blood pressure status (hypertension and normotension)

## Discussion

In our study, periodontitis was associated with an increased risk of BPH in both crude and adjusted analyses; periodontal disease had no independent relationship with BPH in crude analysis, but statistical significance appeared after adjustments and subgroup analysis. Moreover, when we performed sensitivity analyses using the greedy matching method (matched age, BMI, and blood pressure status), the association of periodontal disease with BPH remained the same.

Investigating the association between periodontal disease and BPH risk is an interesting topic. In 2013, Boland et al. [23] performed a case-control study based on USA patients and found that periodontitis (diagnosed according to the ICD-9, including gingivitis) can increase the risk of BPH (with or without urinary obstruction) by 1.50 times (*OR* = 1.50, 95% CI: 1.05–2.10) in males younger than 70 years old after adjustments. Then, they reported subgroup results for Asians (*OR* = 2.90, 95% CI: 2.35–3.71), individuals of European descent (*OR* = 1.20, 95% CI: 1.02–1.30), a Hispanic population (OR = 2.20, 95% CI: 2.04–2.42), an unknown ethnicity group (*OR* = 1.40, 95% CI: 1.29–1.63), and others (*OR* = 3.80, 95% CI: 3.03–4.67). Our results, based on Asians, confirmed their findings, revealing a significant positive association between the two diseases (*OR* = 1.68, 95% CI: 1.26–2.24).

Our study revealed that periodontal disease was probably an independent risk factor for BPH. BPH is strongly associated with age [29], as are the increased prevalence and severity of periodontal disease [30]. Hypertension and diabetes were also associated with BPH in 1966 [31]; accordingly, metabolic syndrome (MetS) patients had significantly larger total PV (+ 1.8 ml, 95% CI: 0.74–2.87, *P* < 0.001) than those without MetS. Boland and colleagues [23] also reported the relationship between BPH and diabetes (*OR* = 1.4, 95% CI: 1.20–1.55), hypertension (*OR* = 1.3, 95% CI: 1.10–1.60), obesity (*OR* = 1.3, 95% CI: 0.91–1.89), and lipid conditions (*OR* = 1.2, 95% CI: 0.96–1.57). These comorbidities might increase the risk of BPH among people in the United States [31]. In our study, the sensitivity analysis matched for age, BMI, and hypertension status revealed that periodontal disease was associated with an increased risk of BPH. Hence, we concluded that the association between periodontal disease and BPH was not influenced by age, hypertension status, BMI or ethnicity. MetS is a cluster of medical conditions, including obesity, impaired glucose metabolism (abnormal glucose metabolism), hypertriglyceridemia, low HDL-C and hypertension [32]. Hyperlipidemia is also shown to increase the risk of BPH [33], and ESR [34] and BMI [35] might increase PV. Hence, we also adjusted for LDL-C, HDL-C, TGs, TC, and ESR after original adjustments for FBG, hypertension status and BMI. As a result, the main findings were unchanged, indicating their robustness. In addition, we also investigated the relationship between periodontal disease and PV. We divided BPH patients into two groups: PV greater than and equal to or less than 60 g, and the results obtained for the two groups were similar to those for overall cases. However, the risk was higher in the group with PV greater than 60 g. This finding indicated a potential dose-response relationship between periodontal disease and PV in BPH patients. Unfortunately, we failed to perform further analysis on this aspect due to limited data.

The primary mechanism of periodontal disease promoting BPH origination and progression may be associated with inflammation and accompanied by possible influences from oral bacteria. The oral cavity presents a potential reservoir for pathogens, including bacteria and viruses [36, 37], and dental plaque may play an important role in this reservoir, possibly also contributing to periodontal disease [38]. Plausible mechanisms may involve the movement of pathogens and dental plaques from the mouth to the prostate through water drinking or/or capillary vessels. Pathogens and dental plaques can enter capillary vessels and thus trigger systemic inflammatory responses. The prostate is an immune-competent organ with a complex immune system, and tissue damage and chronic tissue healing could result in BPH nodules [39, 40]. Hence, when pathogens and dental plaques travel to a normal prostate, prostatic inflammation may occur; later, BPH would develop. In 2017, Estemalik et al. demonstrated an association between oral pathogens and BPH. In that study, they investigated *Porphyromonasgingivalis (Pg), Prevotella intermedia (Pi), Treponema denticola (Td),* and *Escherichia coli (E. coli)* in expressed prostatic secretions from patients with both periodontal disease and chronic prostatitis or BPH and found at least one oral pathogen in 9 out of 10 BPH patients in their prostatic secretions [24]. This finding suggested that periodontal scaling should be covered in routine healthcare for old males and that BPH therapy should be accompanied by periodontal treatment. In addition, asymptomatic prostate inflammation can worsen lower urinary tract symptoms and urinary flow rate in patients with BPH [41], while periodontal treatment can effectively reduce such risks. Additionally, basic laboratory studies have demonstrated that infections by periodontal pathogens can accelerate some systemic conditions by inciting inflammatory responses and further affecting apoptosis, and the mechanisms have been corroborated in atheroma deposition [42], preterm low birth weight among infants [43], cognitive decline [44] and respiratory infection [45] but not in BPH until now. Consequently, experimental studies should be conducted to further explain the potential mechanisms underlying this association.

Neither smoking nor alcohol consumption status was included in our data, so we could not analyze their potential influences in this study. However, a recent systematic review and meta-analysis based on 44,100 subjects showed no significant association between cigarette smoking and BPH risk for either ex-smokers or current smokers [46]. Another systematic review in 2017 showed marked associations of modest alcohol intake with decreased BPH diagnosis and lower urinary tract symptoms [46]. Hence, we can conclude that our results might not be substantially affected by the lack of data on smoking and alcohol consumption. Nevertheless, our study adopted a retrospective design based on existing data, so fragmentary data might affect final results. For example, the information on prostate H/W/L was not recorded for individuals with normal prostates, leading to the missing data on PV for normal subjects. Other potentially relevant information was also not included, such as tooth loss, denture use and decay missing filling tooth (DMFT). Therefore, the associations of denture use and DMFT with BPH were not analyzed to further explore the potential relationship between dental body defect and BPH. Nonetheless, the sensitivity analysis in our study revealed that dental body defects independently affected BPH susceptibility, which might be attributed to the lack of precise information on the above-mentioned indexes. These limitations should be avoided in further studies.

## Conclusions

To our knowledge, this was the first study on the association between periodontal disease and BPH risk in Chinese people. Our study revealed a significant robust relationship between increased BPH risk and periodontal disease. This relationship may be a consequence of periodontal and prostatic inflammation. According to these results, we recommend that old males pay more attention to their oral health, especially BPH patients developing relevant symptoms. Periodontal treatment, which is easy and inexpensive, should be included in algorithms to predict BPH surgery risk in future studies.

## Data Availability

The datasets used and/ or analyzed during the current study are available from the corresponding author upon reasonable request.

## Abbreviations

BMI: Body mass index
BPH: Benign prostatic hyperplasia
CI: Confidence interval
CPI: Community Periodontal Index
CRP: C-reactive protein
DALYs: Disability-adjusted life years
DBP: Diastolic blood pressure
DMFT: Decay missing filling tooth
E: coli: *Escherichia coli*
ESR: Erythrocyte sedimentation rate
FBG: Fasting blood glucose
GBD: Global burden disease
HDL-C: High-density lipoprotein cholesterol
LDL-C: Low-density lipoprotein cholesterol
MetS: Metabolic syndrome
OR: Odds ratio
Pg: Porphyromonasingivalis
Pi: Prevotella intermedia
PV: Prostate volume
SBP: Systolic blood pressure
TC: Total cholesterol
Td: Treponema denticola
TG: Triglycerides
UA: Uric acid
UN: Urea nitrogen
